# Serum Uric Acid and Non-Alcoholic Fatty Liver Disease in Non-Diabetic Chinese Men

**DOI:** 10.1371/journal.pone.0067152

**Published:** 2013-07-23

**Authors:** Yuanliang Xie, Mengjie Wang, Youjie Zhang, Shijun Zhang, Aihua Tan, Yong Gao, Zhengjia Liang, Deyi Shi, Zhang Huang, Haiying Zhang, Xiaobo Yang, Zheng Lu, Chunlei Wu, Ming Liao, Yu Sun, Xue Qin, Yanling Hu, Li Li, Tao Peng, Zhixian Li, Xiaoli Yang, Zengnan Mo

**Affiliations:** 1 Institute of Urology and Nephrology, First Affiliated Hospital of Guangxi Medical University, Nanning, Guangxi Zhuang Autonomous Region, China; 2 Center for Genomic and Personalized Medicine, Guangxi Medical University, Nanning, Guangxi Zhuang Autonomous Region, China; 3 Medical Examination Center, Fangchenggang First People's Hospital, Fangchenggang, Guangxi Zhuang Autonomous Region, China; 4 Department of Occupational Health and Environmental Health at School of Public Health, Guangxi Medical University, Nanning, Guangxi Zhuang Autonomous Region, China; 5 Department of Ultrasound, First Affiliated Hospital of Guangxi Medical University, Nanning, Guangxi Zhuang Autonomous Region, China; 6 Department of Clinical Laboratory, First Affiliated Hospital of Guangxi Medical University, Nanning, Guangxi Zhuang Autonomous Region, China; 7 Medical Scientific Research Center, Guangxi Medical University, Nanning, Guangxi Zhuang Autonomous Region, China; 8 Department of Hepatobiliary Surgery, First Affiliated Hospital of Guangxi Medical University, Nanning, Guangxi Zhuang Autonomous Region, China; Scientific Directorate, Bambino Hospital, Italy

## Abstract

Increased serum uric acid (SUA) levels may be involved in the development of non-alcoholic fatty liver disease (NAFLD) in men presenting with metabolic syndrome (MetS) and/or insulin resistance. We aimed to determine the independent relationship between SUA and NAFLD in non-diabetic Chinese male population, and to explore the determinants of SUA levels among indexes of adiposity, lipid, and genotypes pertaining to triglycerides metabolism, inflammation, oxidative stress, and SUA concentrations. A total of 1440 men, classified depending on the presence of ultrasonographically detected NAFLD, underwent a complete healthy checkup program. Genotypes were extracted from our previously established genome-wide association study database. After adjusting for age, smoking, drinking, body mass index, homeostasis model assessment of insulin resistance, C-reactive protein, creatinine, alanine aminotransferase (ALT) and components of metabolic syndrome, the odds ratio for NAFLD, comparing the highest with the lowest SUA quartile, was 2.81 (95% confidence interval 1.66–4.76). A stepwise multivariate linear regression analysis (R^2^ = 0.238, *P*<0.001) retained age, waist circumference, serum creatinine, triglycerides, the Q141K variant in *ABCG2* (rs2231142) and NAFLD as significant predictors of SUA levels (all *P*<0.001). Besides, ALT and Met196Arg variant in *TNFRSF1B* (rs1061622) additionally associated with SUA among individuls with NAFLD. Our data suggest that in Chinese men, elevated SUA is significantly associated with NAFLD, independent of insulin resistance and other metabolic disorders, such as central obesity or hypertriglyceridemia. Meanwhile, among subjects with NAFLD, index of liver damage, such as elevated ALT combined with genetic susceptibility to inflammation associated with increased SUA levels.

## Introduction

Nonalcoholic fatty liver disease (NAFLD) comprises a spectrum of pathologic conditions including simple steatosis, nonalcoholic steatohepatitis and cirrhosis, influences approximately 20–30% of the general population and its prevalence is increasing worldwide [1]. In China, with continually increasing pandemic of metabolic disorders, such as obesity, insulin resistant and metabolic syndrome (MetS) [2], NAFLD has also been emerging at an alarming rate and posing a very large proportion of the Chinese population at risk of impending liver diseases in the next decade [3], [4]. NAFLD is commonly associated with obesity and insulin resistance, which per se are closely related to a cluster of other metabolic abnormalities, such as hypertriglyceridemia and hyperuricemia [5].

Recently, mounting evidence suggests that elevated serum uric acid (SUA) frequently associates with the development or progression of NAFLD [6], [7]. Several evidences linking SUA and NAFLD have been provided from large population based study of Chinese and American people [8], [9]. Li et al found that SUA level was significantly associated with NAFLD, independ of age, body mass index (BMI), blood lipids, and fasting plasma glucose [8]. While another study suggested that elevated SUA level associated with the development of cirrhosis and increased serum liver enzymes [9]. Prior epidemiological studies showed that UA is an independent risk factor for cardiovascular diseases [10], [11], and the pathological processes included insulin resistance, oxidative stress, and systemic inflammation [12], [13], which are all considered as important risk factors for the development or progression of NAFLD [14]. In fact, a recent cross-sectional analysis of 10732 adults who participated in the National Health and Nutrition Examination Survey 1988–1994 also demonstrated that elevated uric acid level is independently associated with ultrasound-diagnosed NAFLD, regardless of insulin resistance, components of MetS, and indexes of liver and kidney function [15].

Serum uric acid, balanced between serum uric acid production and excretion, is the end product of purine metabolism by liver [16]. Hyperuricemia is a common finding in patients with metabolic syndrome or its components, such as central obesity and hypertriglyceridemia [17]. An inverse correlation was also noted between insulin resistance and decreased renal uric acid clearance, which is itself associated with elevated SUA [18]. In addition, increased triglycerides synthesis in individuals with metabolic abnormalities would also accelerate SUA production and accumulation [5]. Besides, inflammatory factors, such as tumor necrosis factor α and it induced oxidative and apoptosis stress have been suggested to be important factors for more serious liver damage, resulting in uric acid production. Since raising evidences suggest that chronic elevation of SUA concentration would be a causal factor for diseases, such as metabolic abnormalities and cardiovascular mortality, a well understanding of factors that influence SUA levels in population or in NAFLD patients will provide a more accurate interpretation of SUA-NAFLD relationship and has potential implications on NAFLD treatment in the population. Therefore, the purpose of the present study is 2-fold: (1) to test the hypothesis that SUA-NAFLD interrelationship occurs independently from insulin resistance, MetS, and its components; (2) to explore the determinants of SUA levels among indexes of adiposity, lipid, and genotypes pertaining to triglycerides metabolism, inflammation, oxidative stress, and SUA concentrations in a large series of non-diabetic Chinese men.

## Participants and Methods

### Study Population

All subjects, who participated in a large-scale physical examination from September 2009 to December 2009, were recruited continuously from the Fangchenggang Area Male Healthy and Examination Survey (FAMHES). The study has been described previously in detail [19]. In brief, FAMHES is a population-based epidemiological cohort study in area of Guangxi, China, aiming at investigating the effects of environmental and genetic factors and their interaction on the health of male and the progress of age-related chronic diseases. After excluded subjects who currently diagnosed with diabetes mellitus, coronary heart disease, stroke, hyperthyroidism, rheumatoid arthritis, and cancer or taking any kind of medication within four weeks, or with impaired hepatic and renal function, 2426 subjects aged 20–69 years were included. Of those eligible, subjects with incomplete data involved ultrasonography (n = 212), blood test values (n = 342), and genotype of the whole genome (n = 83), or with hepatitis B infection (n = 268), alcohol consumption >40 g/day and >5 times/week (n = 52), or C-reactive protein (CRP) value higher than 10 mg/l (n = 29) were further excluded to avoid bias. Finally, 1440 men with complete data were included for analyses. All subjects provided written informed consents, and the study was approved by Ethics and Human Subject Committee of Guangxi Medical University.

### Data Collection

Participants in the FAMHES underwent a detailed medical interview that included information on demographics, medical history, smoking status and alcohol consumption. Current smokers were defined as smoking at least once a day and lasting for more than six months. Alcohol consumption was defined as consumption of alcoholic drinks (beer, wine, or hard liquor) once or more per week. Anthropometric parameters, including height, weight, waist circumference (WC), and blood pressure were measured by trained personnel using a standardized protocol [20]. BMI was calculated as weight in kilograms divided by the square of height in meters. Fasting blood samples were drawn between 8 a.m. and 10 a.m. Serum low-density lipoprotein cholesterol (LDL-C), high-density lipoprotein cholesterol (HDL-C), triglycerides, fasting blood glucose (FBG), alanine aminotransferase (ALT), serum creatinine and SUA were measured using a Dimension-RxL Chemistry Analyzer (Dade Behring, Newark, DE, USA). Insulin was measured using COBAS 6000 system E601 electrochemiluminescence immunoassay (Roche Diagnostics, IN, Germany), and high-sensitivity CRP level was detected using the immunoturbidimetric assay on the Hitachi 7600 autoanalyzer (Hitachi Corp, Tokyo, Japan)

### Ultrasonography

Two experienced ultrasonographers assessed for liver size, contour, echogenicity, structure and posterior beam attenuation. Fatty liver was diagnosed based on the findings of abdominal ultrasonography using a portable ultrasound device (GE, LOGIQ e, 5.0-MHz transducer, USA) and included the presence of increased liver echogenicity (bright), and stronger echoes in the hepatic parenchyma than in the renal parenchyma, vessel blurring and narrowing of the lumen of the hepatic veins [21], [22].

### Definition of disease

The diagnosis of NAFLD was based on abdominal ultrasound without including alcohol consumption, viral, or autoimmune liver disease [23]. Men with a SUA level >420 µmol/L was defined as hyperuricemia [24]. Insulin resistance was assessed through the homeostasis model assessment algorithm using the following established formulas: glucose (mmol/liter)×Insulin (mlU/liter)/22.5, and a value of 2.4 or higher was considered insulin resistant [25]. The metabolic syndrome was diagnosed using the 2005 National Cholesterol Education Program-Adult Treatment Panel III (NCEP-ATP III) criteria for Asian Americans [26]. The NCEP-ATP III has defined the metabolic syndrome as the presence of three or more of the five characteristics of (1) waist circumference ≥90 cm; (2) triglycerides ≥1.7 mmol/L, (3) HDL-C <1.03 mmol/L, (4) blood pressure ≥130/85 mm Hg or current use of antihypertensive medications, and (5) fasting blood glucose ≥5.6 mmol/L or previous diagnosis of type 2 diabetes mellitus or use of oral antidiabetic agents or insulin.

### DNA analysis

We extracted several polymorphisms related to lipid metabolism (rs738409 in *PNPLA3*) [27], inflammation (rs1800629 in *TNFα*, rs1061622 in *TNFRSF1B, and* rs8192284 in *IL6Rα*) and oxidative stress (rs887829 in *UGT1A1 and* rs4880 in *SOD2*) that had been previously found to be associated with metabolic disorders such as obesity and NAFLD [28], [29], [30], and variants involved in uric acid concentrations (rs2231142 in *ABCG2*, rs1165205 in *SLC17A3*, missense rs16890979 in *SLC2A9*) [31], [32], in our previously established genome-wide association database [19]. The genotyping methods have been described previously [19].

### Statistical Analysis

We classified participants on the basis of quartiles of serum uric acid, with data presented as mean ± SE. For descriptive analyses across the quartile group of SUA, we performed chi-square analyses for categorical variables and ANOVA for continuous traits. Logistic regression analyses were use to assess the association of NAFLD, MetS or its components with SUA, and results were presented by odds ratio (OR) and 95% confidence intervals (CI). A multivariate linear regression analysis was used to determine the effects of anthropometric, clinical, metabolic, and genetic variants on the logarithm of SUA concentrations. For maintaining the symmetry and comparability of per-unit-effect estimates, all models presented use log-transformed values of blood variables. All statistical analyses were performed with PASW Statistics 18 (Chicago, IL, USA). Statistical tests were 2-sided, and a *P* value<0.05 was considered statistically significant.

## Results

### Patient Characteristics

Among the 1440 participants, the median age of the study was 36 years (interquartile range, 29–44), and median SUA was 374.0 µmol/L (interquartile range, 326.3–426.0). The prevalence of hyperuricemia, NAFLD and MetS were 27.6% (n = 398), 26.3% (n = 379) and 14.0% (n = 202), respectively. Table 1 shows the characteristics of study subjects according to quartile of SUA. Participants with higher serum uric acid concentrations exhibited higher prevalence of NAFLD and MetS. Meanwhile, BMI, waist circumference, systolic and diastolic blood pressure, LDL-C, triglycerides, fasting insulin, HOMA-IR, ALT, and serum creatinine were significantly higher, while HDL-C was lower, among men with higher SUA levels.

**Table 1:** Characteristics of participants according to quartile (Q) of serum uric acid (n = 1440).

	Serum uric acid quartile				
Variable	Q1 (n = 360)	Q2 (n = 363)	Q3 (n = 366)	Q4 (n = 351)	*P* value
Uric acid (µmol/L)	288.3±1.8	350.6±0.7	400.2±0.8	490.4±3.0	<0.001
Age (years)	38.8±0.6	37.2±0.6	37.0±0.6	37.5±0.6	0.113
Current smoker (n, %)	177 (49.2)	184 (50.7)	186 (50.8)	170 (48.4)	0.899
Alcohol drinker (n, %)	297 (82.5)	312 (86.0)	317(86.6)	305 (86.9)	0.31
NAFLD (n, %)	36 (10.0)	71 (19.6)	120 (32.8)	152 (43.3)	<0.001
BMI (kg/m^2^)	22.2±0.1	22.9±0.2	23.6±0.2	24.9±0.2	<0.001
Waist circumference (cm)	77.7±0.4	79.5±0.5	81.5±0.5	85.3±0.5	<0.001
Systolic blood pressure (mmHg)	117.6±0.8	117.0±0.7	118.3±0.8	121.0±0.8	0.003
Diastolic blood pressure (mmHg)	75.7±0.5	75.6±0.5	77.7±0.5	79.5±0.6	<0.001
LDL-C (mmol/L)	2.87±0.04	2.97±0.04	3.00±0.04	3.15±0.04	<0.001
HDL-C (mmol/L)	1.44±0.02	1.41±0.02	1.36±0.02	1.33±0.02	<0.001
Triglycerides (mmol/L)	1.27±0.08	1.45±0.10	1.61±0.09	2.04±0.09	<0.001
Glucose (mmol/L)	5.36±0.06	5.27±0.05	5.29±0.05	5.33±0.03	0.589
Metabolic syndrome (n, %)	25 (6.9)	35 (9.6)	52 (14.2)	90 (25.6)	<0.001
Insulin (mlU/L)	6.6±0.3	7.2±0.3	8.1±0.3	10.4±0.5	<0.001
HOMA-IR	1.6±0.1	1.7±0.1	1.9±0.1	2.5±0.1	<0.001
C-reactive protein (mg/L)	0.93±0.08	0.96±0.07	1.03±0.07	1.15±0.07	0.152
ALT (IU/L)	39.4±1.03	42.0±1.07	47.9±1.44	53.9±1.55	<0.001
Creatinine (µmol/L)	82.2±0.6	85.4±0.6	89.2±0.6	92.7±0.7	<0.001

Data are means ± SE or raw numbers (%). Continuous data were used for univariate general linear models and categorical data were analyzed by χ^2^ tests.

Abbreviation: ALT, alanine aminotransferase; BMI, body mass index; LDL-C, serum low-density lipoprotein cholesterol; HDL-C, serum high-density lipoprotein cholesterol; HOMA-IR, homeostasis model assessment of insulin resistance; NAFLD, nonalcoholic fatty liver disease.

### Association between Serum Uric Acid Concentrations and NAFLD, MetS or its Components

The ORs for NAFLD increased progressively across the SUA quartiles (all *P*<0.001 for trend) (Table 2). After adjusting for age smoking, and drinking (model 1), the OR for NAFLD, comparing the highest with the lowest SUA quartile, was 7.51 (95% CI 4.98–11.31). Further adjustment for BMI (model 2) substantially attenuated the magnitude of the ORs for NAFLD, but did not affect statistical significance. Using the lowest SUA quartile as reference, the ORs for NAFLD was 1.95 (95% CI 1.16–3.31), 3.08 (95% CI 1.85–5.14), and 2.81 (95% CI 1.66–4.76) for quartiles 2, 3, and 4, respectively (*P*<0.001 for trend), after further adjusting for HOMA-IR, CRP, creatinine, ALT and components of metabolic syndrome (model 5).

**Table 2:** Odds ratios and 95% confidence interval for NAFLD, metabolic syndrome, and its components according to quartile (Q) of serum uric acid.

	Serum uric acid				
	Q1	Q2	Q3	Q4	*P* for trend
NAFLD					
Model 1	1.00	2.34 (1.51–3.61)	4.79 (3.17–7.24)	7.51 (4.98–11.31)	<0.001
Model 2	1.00	1.84 (1.13–3.02)	3.17 (1.99–5.07)	3.35 (2.10–5.34)	<0.001
Model 3	1.00	1.85 (1.13–3.03)	3.16 (1.98–5.06)	3.30 (2.07–5.28)	<0.001
Model 4	1.00	1.85 (1.11–3.07)	3.17 (1.94–5.17)	3.32 (2.01–5.49)	<0.001
Model 5	1.00	1.95 (1.16–3.31)	3.08 (1.85–5.14)	2.81 (1.66–4.76)	<0.001
Metabolic syndrome					
Model 1	1.00	1.57 (0.91–2.70)	2.49 (1.49–4.14)	5.32 (3.28–8.62)	<0.001
Model 2	1.00	1.05 (0.58–1.92)	1.32 (0.75–2.31)	2.01 (1.17–3.44)	0.019
Model 3	1.00	1.06 (0.58–1.94)	1.31 (0.74–2.30)	2.10 (1.22–3.63)	0.012
Model 4	1.00	1.03 (0.56–1.89)	1.26 (0.71–2.25)	1.98 (1.12–3.50)	0.031
Components of metabolic syndrome*					
Central obesity	1.00	1.03 (0.49–2.15)	1.43 (0.72–2.86)	2.01 (1.00–4.03)	0.101
Hypertriglyceridemia	1.00	1.51 (0.99–2.31)	1.74 (1.15–2.65)	3.11 (2.03–4.77)	<0.001
Elevated BP	1.00	1.09 (0.76–1.56)	1.23 (0.86–1.77)	1.09 (0.74–1.59)	0.703
Low HDL cholesterol	1.00	0.48 (0.25–0.93)	0.72 (0.39–1.33)	0.64 (0.34–1.21)	0.181
Hyperglycemia	1.00	0.85 (0.59–1.22)	0.73 (0.50–1.06)	0.77 (0.52–1.15)	0.402

Model 1 was adjusted for age smoking, and drinking;

Model 2 was further adjusted for BMI;

Model 3 was further adjusted for HOMA-IR and C-reactive protein;

Model 4 was further adjusted for serum creatinine and alanine aminotransferase;

Model 5 was further adjusted for the components of metabolic syndrome (variables as categories).

* Fully adjusted model without component itself.

Serum levels of HOMA-IR, C-reactive protein, creatinine and alanine aminotransferase were log transformed.

Abbreviation: BP, blood pressure; NAFLD, nonalcoholic fatty liver disease.

The ORs for metabolic syndrome substantially increased with increasing concentrations of SUA (Table 2). Compared with individuals in the lowest SUA quartile, those in the highest quartile had an OR of 1.98 (95% CI 1.12–3.50) in the full multivariate model (model 4). Further adjusted for components of MetS (without component itself), SUA was positively associated with hypertriglyceridemia (OR = 3.11, 95% CI 2.03–4.77) (*P*<0.001 for trend), and central obesity (OR = 2.01, 95% CI 1.00–4.03) with a borderline statistical significance (*P* = 0.05).

**Figure 1:**
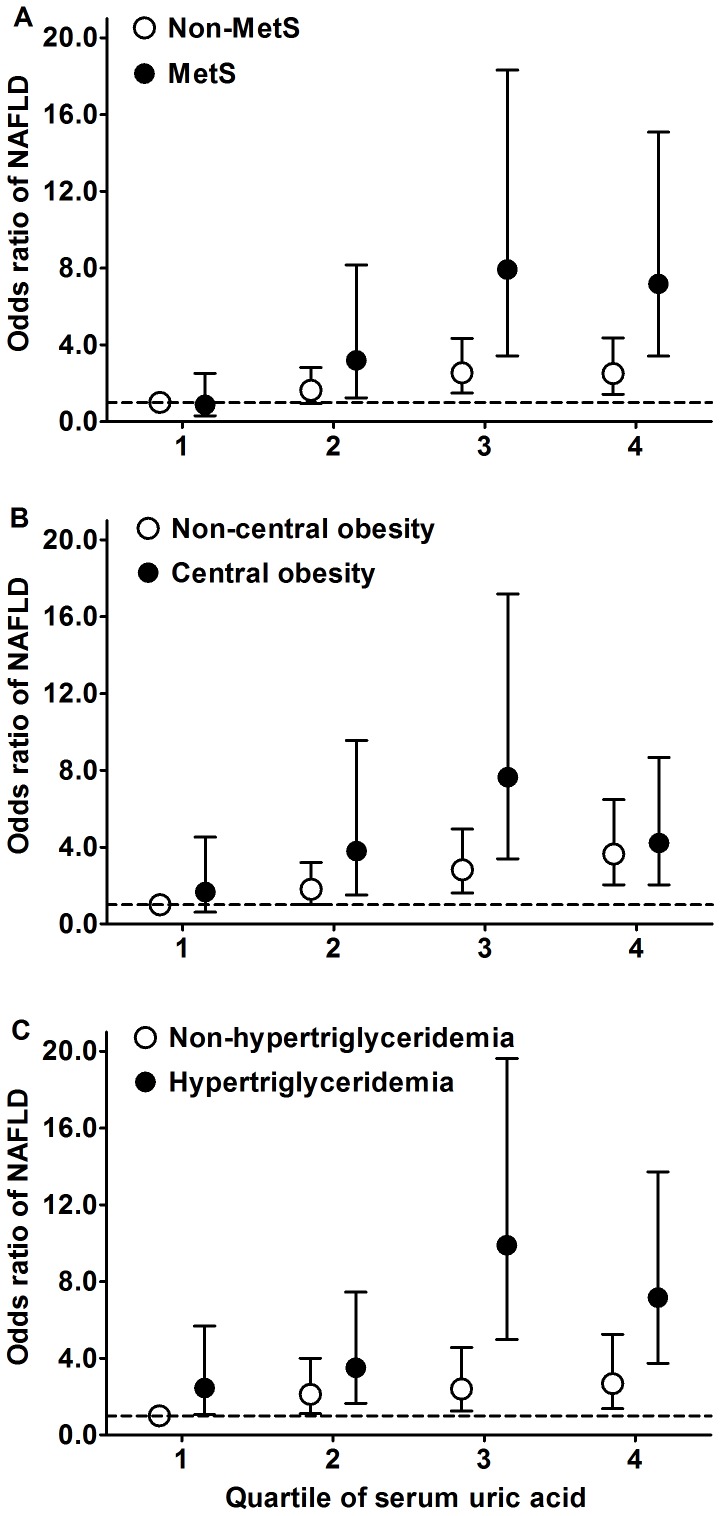
Odds ratios (OR) and 95% confidence interval (CI) for NAFLD. Adjusted for age, smoking, drinking, BMI, HOMA-IR, C-reactive protein, creatinine and alanine aminotransferase The black and white circles are the ORs for NAFLD among subjects with or without MetS (A), central obesity (B), and hypertriglyceridemia (C) respectively. The error bars indicate the 95% CI of OR, and broken lines indicate the OR = 1. Serum levels of HOMA-IR, C-reactive protein, creatinine and alanine aminotransferase were log transformed.

We also duplicated our analysis among subgroups with or without MetS, central obesity, or hypertriglyceridemia (Figure 1). In general, the risks of NAFLD were more pronounced among subjects with higher concentration of SUA and with combination of metabolic disorders. Among the subjects without metabolic syndrome (Figure 1A), compared with the lowest serum uric acid quartile (reference group), those in the highest quartile had an OR of 2.50 (95% CI 1.43–4.36) for NAFLD. The OR increased noticeably with the combination of MetS and high serum uric acid concentrations with an OR of 7.18 (95% CI 3.42–15.08) for NAFLD. No significant interaction was observed between serum uric acid and MetS (*P* for interaction = 0.36). In addition, participants with central obesity (Figure 1B) and hypertriglyceridemia (Figure 1C), compared with the reference group, those in the highest quartile had ORs of 4.22 (95% CI 2.05–8.67) and 7.16 (95% CI 3.74–13.72) for NAFLD respectively. No significant interaction was observed between NAFLD and central obesity, and hypertriglyceridemia (*P* for interaction = 0.22, and 0.94 respectively).

### Factors Associated With Serum Uric Acid Concentrations

**Figure 2:**
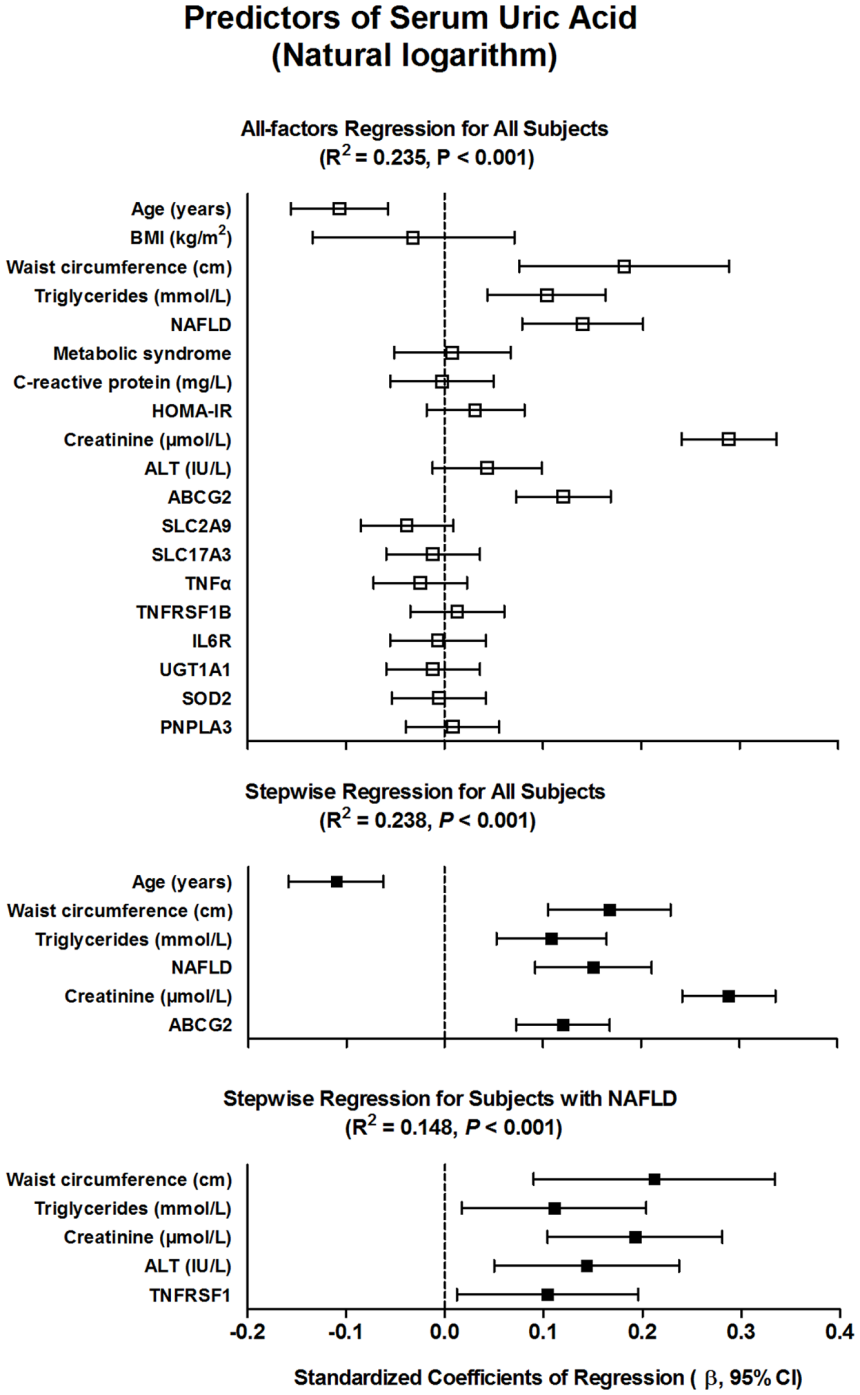
Multiple linear regression analysis of the logarithm of serum uric acid. The squares are the standardized regression coefficients (β) and the error bars indicate the 95% CI of β, and broken lines indicate the β coefficients = 0. Genomic variants were coded as dummy variables: 0 for homozygosity for wild-type alleles, 1 for heterozygosity, and 2 for homozygosity for effect alleles.

Ln-normalized SUA level was introduced as a dependent variable in the multivariate linear regression models (Figure 2), using age, BMI, waist circumference, NAFLD and MetS (both classified as yes or no), and log-transformed values of triglycerides, CRP, HOMA-IR, creatinine, and ALT, and the genomic variants as independent variable. Overall, this model explained 23.5% of the variability in logarithm of SUA concentrations. Among all subjects, a final constructed model using a stepwise method (probability to enter ≤0.05; to remove ≥0.10), found age (β = −0.11, 95% CI −0.16 to −0.06), WC (β = 0.17, 95% CI 0.11–0.23), NAFLD (β = 0.15, 95% CI 0.09–0.21), log-transformed serum creatinine (β = 0.29, 95% CI, 0.24–0.34) and triglycerides (β = 0.11, 95% CI 0.05–0.16), as well as the Q141K variant in *ABCG2* gene (β = 0.12, 95% CI 0.07–0.17) as significant predictors (all *P*<0.001) of the logarithm of SUA levels (R^2^ = 0.238, *P*<0.001). When duplicated our stepwise regression analysis among subjects with NAFLD, interestingly, the WC (β = 0.21, 95% CI 0.09–0.33, *P* = 0.001), log-transformed triglycerides (β = 0.11, 95% CI 0.02–0.20, *P* = 0.02), creatinine (β = 0.19, 95% CI 0.10–0.28, *P*<0.001), and ALT (β = 0.14, 95% CI 0.05–0.24, *P* = 0.003), and the Met196Arg variant in *TNFRSF1B* gene (β = 0.10, 95% CI 0.01–0.20, *P* = 0.027) were positively associated with logarithm of SUA concentrations (R^2^ = 0.148, *P*<0.001).

## Discussion

We observed a strong positive association between elevated serum uric acid levels and the risks of NAFLD in non-diabetic Chinese men, independent of insulin resistance or metabolic syndrome status. Our data implied the crucial role of SUA as an important independent risk factor for NAFLD. In addition, we identified a missense SNP in *ABCG2* gene (rs2231142) associated with SUA level, further clustered with independent variables, such as age, waist circumference, NAFLD, creatinine and triglycerides, which explained 23.8% of the variability in the logarithm of serum uric acid concentration. Besides, ALT and variant Met196Arg of *TNFRSF1B* gene (rs1061622) combined with WC, creatinine, and triglycerides, associated with SUA among subjects with NAFLD. Our findings are partly consistent with and extend an earlier Chinese cross-sectional study [8], which reported lower prevalence of hyperuricemia (14.7% vs. 27.6%) and NAFLD (11.8% vs. 26.3%) than our results. A possible explanation for the different prevalence may originate from the characteristics of the studied populations. In the present study, Fangchenggang people living in coastal areas have a relatively frequent seafood diet, which are closely related with elevated serum uric acid [33], and possibly influence the natural characteristics of NAFLD.

Studies have consistently shown an association between elevated SUA and risk of NAFLD, and are in accord with prior hypotheses suggesting that SUA might be an important contributor to the development of NAFLD. In studies of 8925 employees of Ningbo province in China, hyperuricemia was related to NAFLD, independently of metabolic risk factors at baseline, and after a 3-year follow-up, SUA levels were independently and positively associated with the risk for incident NAFLD, although insulin resistance was not considered [6], [8]. Another prospective study among healthy Korean men also found SUA appeared to be an independent predictor for developing ultrasonographically detected NAFLD; the investigators did not measure the waist circumference, which may be a better surrogate marker of central obesity [7]. Our data combined with previous findings suggested that higher levels of SUA are commonly associated with metabolic syndrome and its five components, especially central obesity and hypertriglyceridemia [17], which are tightly related to NAFLD [34]. In addition, we also noted a strong positive association between higher levels of SUA and the risks of NAFLD, independent of indexs of obesity, insulin resistance, MetS, and liver and kidney function. Although SUA increase is also observed in individuals with insulin resistance, we found that the increased risks for NAFLD by hyperuricemia could not be explained merely through peripheral HOMA-IR. The possible explanation is that SUA increase is individuals with insulin resistance, probably because hyperinsulinemia would cause lower renal UA excretion [35], and indirect act on SUA via reduction of adipocyte sensitivity to insulin and then increases triglyceride lipolysis within adipose depots [5].

Metabolic and renal factors and genetic variation might contribute to determining uric acid concentration through regulation of uric acid synthesis, excretion, or reabsorption [36]. Because whether SUA as a marker or a cause or both, strategies that aim at monitoring or decreasing SUA levels may have clinical beneficial effects to prevent or reduce the risk of NAFLD. Our present result also suggested that the Q141K variant in *ABCG2*, leads to variable degree of SUA concentration in men, in conceptual agreement with the ABCG2's function of altering uric acid transport in kidney proximal tubule cell and excretion in liver via the biliary system [37]. However, more importantly, the relatively strong association of increased SUA levels with NAFLD raises the possibility that SUA overload might play some pathogenic role in the development of NAFLD, given that progressive SUA accumulation contributes to inflammatory and oxidative effects [12], [13]. On the contrary, these levels are not influenced by global obesity, MetS, CRP, HOMA-IR, ALT or by genomic variants related to PNPLA3, chronic inflammation, and oxidative stress in the overall population.

Recently, evidence revealed that uric acid, released from injured cells, induced sterile inflammation [38], [39]. Small molecules like ATP or large crystals like UA can be transformed or exported from the liver under normal physiological condition. However, non-alcoholic steatohepatitis, one of important component of liver damage, might induce cell death results in the release and accumulation of molecules are not present in the extracellular environment during health, such as UA [40]. Thus, our results of positive association of elevated serum ALT and the Met196Arg variant in *TNFRSF1B* with SUA concentrations among subjects with NAFLD further support this proposal, which is called damage associated molecular patterns (DAMPs), and suggested that the release of UA might be accelerated when tissue injury (combined with a genetic susceptibility to inflammation) happened in NAFLD patients [41]. Then UA as a promising production of DAMPs triggers sterile inflammation and increases organ damage, based on prior hypothesis role of inducing inflammation and oxidative stress.

We simultaneously investigate the effects of metabolic syndrome and its components, and insulin resistance on the relationship between SUA and NAFLD in a large population-based sample. And to our knowledge, we first explore the determinants of SUA levels among multiple variables and genotypes in Chinese men. However, several potential limitations are admitted. First, NAFLD diagnosis is based on ultrasound imaging, which is neither sensitive enough to distinguish hepatic steatosis from NASH, nor to distinguish the stage of hepatic fibrosis only in the case that cirrhosis is present. However, ultrasonographic examination currently remains the primary method for epidemiologic studies of NAFLD owing to its non-invasiveness, safety, wide availability and convenience. Second, given the nature of cross-sectional study, whether elevated SUA is a cause or an effect of NAFLD cannot be answered accurately. Furthermore, a recent study showed that hyperuricaemia was independently associated with severity of steatosis among chronic hepatitis C patients; therefore, another potential limitation might be including patients with hepatitis C virus infection, due to the absence of diagnostic markers in our study.

In conclusion, elevated serum uric acid is independently associated with NAFLD regardless of insulin resistance and metabolic syndrome status, especially hypertriglyceridemia or central obesity. SUA are interrelated with age, waist circumference, NAFLD, creatinine, triglycerides, and the Q141K variant in *ABCG2* in non-diabetic Chinese men. Meanwhile, among subjects with NAFLD, index of liver damage, such as elevated ALT combined with genetic susceptibility to inflammation (Met196Arg variant in *TNFRSF1B*) associated with increased SUA levels. Strategies that aim at modulating the SUA levels and/or improving liver function may have significant clinical implications for the prevention and treatment of NAFLD.
